# Ground State Energy of Dense Gases of Strongly Interacting Fermions

**DOI:** 10.1007/s00023-024-01506-2

**Published:** 2024-11-05

**Authors:** Søren Fournais, Błażej Ruba, Jan Philip Solovej

**Affiliations:** Department of Mathematical Sciences, University of Copenhagen, Universitetsparken 5, 2100 Copenhagen, Denmark

## Abstract

We study the ground state energy of a gas of *N* fermions confined to a unit box in *d* dimensions. The particles interact through a two-body potential with strength scaled in an *N*-dependent way as $N^{-\alpha }v$, where $\alpha \in \mathbb {R}$ and *v* is a function of positive type satisfying a mild regularity assumption. Our focus is on the strongly interacting case $\alpha <1-\frac{2}{d}$. We contrast our result with existing results in the weakly interacting case $\alpha >1-\frac{2}{d}$ and the transition happening at the mean-field scaling $\alpha =1-\frac{2}{d}$. Our proof is an adaptation of the bosonization technique used to treat the mean-field case.

## Introduction

One important goal in mathematical physics is to rigorously establish accurate approximations to ground energies of gases of quantum particles. For Fermi gases of low density $\rho$, this was initiated in [1], where the leading contribution from the interactions to the energy was obtained. For gases in three-dimensional space, it is of order $\rho ^2$ and comes entirely from the potential between pairs of fermions with opposite spin. The same result with better error bounds (but stronger regularity assumptions on the potential) was derived in [2, 3]. The currently best known upper bound [3] has errors of order $\rho ^{\frac{7}{3}}$, hence comparable with the conjectural next term [4]. In the case of spinless (or spin-polarized) fermions, already the leading contribution of the interactions is expected to be of order $\rho ^{\frac{8}{3}}$. Using the method of cluster expansions this expected form of the interaction energy was established in [5] as an upper bound. The method was also extended in [6] to cover fermions with spin, leading to an upper bound with errors almost as small as in [3], but holding for a much larger class of potentials. There are also interesting results about gases at positive temperature [7, 8] and about one-dimensional gases [9].

There seems to be much less work on dense gases, presumably mainly due to the mathematical difficulty of the problem. Three early works on charged systems were [10–12]. In particular, [10] dealt with the thermodynamic limit of the Jellium model. It has become popular to instead study systems of increasingly many particles confined to a fixed volume, and we also do this in the present work. This may be deemed somewhat unphysical, but it is not implausible that some conclusions of such investigations remain valid in more realistic models. Besides, simplified models provide a useful playground to develop new mathematical methods.

We consider the system of *N* non-relativistic, interacting spinless fermions on the *d*-dimensional torus ${\mathbb {T}}^d = ({\mathbb {R}} / {\mathbb {Z}})^d$ of dimension $d \ge 2$. We assume that interactions between particles are described by a two-body potential *v* whose magnitude is modulated by the factor $N^{- \alpha }$, where $\alpha$ is a fixed numerical parameter. That is, we study the Hamiltonian

1 $$\begin{aligned} H_N = \sum _{i=1}^N (-\Delta _i) + N^{-\alpha } \sum _{1 \le i < j \le N} \hspace{-0.75em} v(x_i-x_j) \end{aligned}$$

on the Hilbert space ${\mathcal {H}}_N=L^2_a(({\mathbb {T}}^{d})^N)$ of square-integrable functions antisymmetric under permutations of the *N* copies of ${\mathbb {T}}^d$.

The lack of spin degrees of freedom in (1) does not make as much of a difference as for dilute gases, and we make this assumption mostly to simplify the presentation. In Sect. 2, we comment how the statement of our results should be modified to incorporate spin.

Since the (*N*-independent) length scales determined by the interaction are much larger than $N^{- \frac{1}{d}}$, which is the expected typical distance between the nearest particles, we are studying a very dense system.

The orders of magnitude of the two terms in (1) are $N^{1+ \frac{2}{d}}$ and $N^{2- \alpha }$, respectively. This naive comparison suggests that the interaction potential is a small perturbation for $\alpha > 1 - \frac{2}{d}$ and that it plays a more dominant role for $\alpha < 1 - \frac{2}{d}$. The intermediate choice $\alpha = 1 - \frac{2}{d}$ is often called the mean-field scaling.

Here, we consider the ground state energy $E_N$ (the infimum of the spectrum of $H_N$). In order to frame the further discussion, let us mention two elementary bounds on $E_N$.

### Proposition 1

Suppose that the Fourier transform ${\widehat{v}}: (2 \pi \mathbb {Z})^d \rightarrow \mathbb {R}$ of *v* satisfies

2 $$\begin{aligned} {\widehat{v}}(k) = {\widehat{v}}(-k) \ge 0 \text { for } k \ne 0, \qquad \sum _{k \in (2 \pi \mathbb {Z})^d} \hspace{-0.75em} |k| {\widehat{v}}(k) < \infty . \end{aligned}$$

Introduce the quantity

3 $$\begin{aligned} E_N^{(0)} = \min _{\begin{array}{c} p_1,\dots ,p_N \in (2 \pi {\mathbb {Z}})^d \\ \text {distinct} \end{array}} \sum _{i=1}^N |p_i|^2 + \frac{N^{2 - \alpha }}{2} \int _{{\mathbb {T}}^d} v - \frac{N^{1- \alpha }}{2} v(0). \end{aligned}$$

The minimum $E_N$ of the spectrum of the operator (1) satisfies

4 $$\begin{aligned} E_N^{(0)} \le E_N \le E_N^{(0)} + c N^{1 - \alpha - \frac{1}{d}} \sum _{k \in (2 \pi \mathbb {Z})^d} \hspace{-0.75em} |k| {\widehat{v}}(k). \end{aligned}$$

for some positive constant *c*.

The elementary bounds (4) determine the ground state energy up to an error of size $O(N^{1-\alpha - \frac{1}{d}})$. The upper bound in (4) is the expectation value $\langle \psi _0 | H_N \psi _0 \rangle$ in a ground state $\psi _0$ of the non-interacting gas. We notice that $E_N^{(0)}$ differs from this non-interacting expectation value $\langle \psi _0 | H_N \psi _0 \rangle$ by an explicit term of order $N^{1-\alpha -\frac{1}{d}}$. In this work, we show that for strong interactions, i.e. $\alpha < 1 - \frac{2}{d}$, we have $E_N = E_N^{(0)}+ o(N^{1-\alpha - \frac{1}{d}})$. Hence, we show a correction to the non-interacting approximation of order $N^{1-\alpha -\frac{1}{d}}$.

We remark that if we replace ${\mathbb {T}}^d$ by ${\mathbb {T}}^d_L = ({\mathbb {R}} / L {\mathbb {Z}})^d$, $N^{- \alpha }$ in (1) by 1, and take *v* to be the *L*-periodization of $V \in L^1({\mathbb {R}}^d)$ with Fourier transform satisfying ${\widehat{V}}(k) \ge 0$ and $\int _{{\mathbb {R}}^d} |k| {\widehat{V}}(k) < \infty$, bounds analogous to (4) give, in the limit $N \rightarrow \infty$, $L \rightarrow \infty$ with fixed $\rho = \frac{N}{L^d}$,

5 $$\begin{aligned} \liminf \frac{E_N}{L^d}&\ge c_1 \rho ^{1 + \frac{2}{d}} + \frac{\rho ^2}{2} \int _{{\mathbb {R}}^d} V(x) dx - \frac{\rho }{2} V(0), \end{aligned}$$

6 $$\begin{aligned} \limsup \frac{E_N}{L^d}&\le c_1 \rho ^{1 + \frac{2}{d}} + \frac{\rho ^2}{2} \int _{{\mathbb {R}}^d} V(x)dx - \frac{\rho }{2} V(0) + c_2 \rho ^{1 - \frac{1}{d}} \int _{{\mathbb {R}}^d} |k| {\widehat{V}}(k) dk , \end{aligned}$$

with explicit constants $c_1,c_2$ depending only on *d*. To the best of our knowledge, these are the best known bounds on the thermodynamic energy density for large particle density.

The upper bound in (4) may be seen as the first order of the perturbative expansion. A rigorous version of the perturbative expansion up to second order has been discussed in [13], which focuses on $d=3$ and the mean-field scaling $\alpha = \frac{1}{3}$. The result of [13] applies only to small enough *v*. Accuracy of the expansion is better in the regime of weak interactions $\alpha > \frac{1}{3}$. In this case, the proofs in [13] show that, at least for regular enough *v*,

7 $$\begin{aligned} E_N = \langle \psi _0 | H_N \psi _0 \rangle + O (N^{1- 2\alpha }). \end{aligned}$$

In fact, up to $o(N^{1- 2 \alpha })$ errors, $E_N- \langle \psi _0 | H_N \psi _0 \rangle$ is given by an explicit formula. Notice that $N^{1 - 2 \alpha } \ll N^{1 - \alpha - \frac{1}{d}} = N^{\frac{2}{3}-\alpha }$. In this sense the upper bound in (4) is sharp up to terms of lower order in *N* in the case $d=3$, and weak interactions $\alpha >\frac{1}{3}$.

Further progress in understanding the ground state energy for $d=3$, $\alpha = \frac{1}{3}$ was made in two series of works, [14–16] and [17, 18]. In [14], a new method of approximate bosonization (inspired by earlier works in physics literature, e.g. [19–25]) was developed to derive an upper bound on $E_N$. The bound was (at that time conjecturally) sharp up to $O(N^{\frac{1}{3}-\epsilon })$ errors, with some explicit $\epsilon >0$. The result was proved for potentials with positive and compactly supported Fourier transform. The first matching lower bound on $E_N$ was obtained in [15], where it was also assumed that the Fourier transform of the potential has small $\ell ^1$ norm. A generalization to potentials as in Proposition 1 was obtained in [16]. Around the same time, a slightly different approach to the bosonization method was proposed in [17], leading to similar results for the ground state energy. This new approach was further developed in [18], where an upper bound for the ground state energy was derived for square-integrable potentials. This was a significant improvement because it allowed for the treatment of the Coulomb potential. Some excited states of the gas were also considered in [17, 26].

The works [14–17, 26] show in particular that in the mean-field scaling (at least for $d=3$) neither the lower nor the upper bound in (4) is sharp up to $o(N^{1- \alpha - \frac{1}{d}})$. By contrast, the upper bound is sharp for $\alpha > 1 - \frac{2}{d}$. The goal of this work is to complete this simple picture by showing that for strongly interacting systems, characterized by $\alpha < 1 - \frac{2}{d}$, the lower bound is sharp. We state this precisely in Theorem 2, which is our main result. We note that our result shows that the trial state $\psi _0$ is not good enough to recover the ground state energy up to errors much smaller than $N^{1- \alpha - \frac{1}{d}}$. Our proof is essentially a construction of better trial states.

The proof of our result is based on the bosonization method inspired by the work in [14–18]. In the bosonization method, the starting point is to perturb around the Slater determinant $\psi _0$ by acting with fermionic operators that annihilate a particle in the Fermi sea and create a particle outside. These operators are combined to form almost bosonic operators quadratic in the fermionic operators. This allows a Hamiltonian quartic in fermions to be related to an operator quadratic in the almost bosonic operators. The almost bosonic operators are constructed in different ways in [14–16] and in [17, 18], but in both approaches a difficulty is to approximate the kinetic energy operator—which is only quadratic in the *fermionic* operators—by a quadratic *bosonic* operator. Quadratic operators in bosons are known to be exactly diagonalizable by Bogoliubov transformations. Implementing these transformations in the almost bosonic setting introduces manageable errors in [14–18]—essentially because the ground state of the effective quadratic Hamiltonian has (in a suitable sense) *O*(1) bosonic particles.

In our setting of strong interactions, a similar approach led us to effective bosonic states with an unmanageable number of particles. However, to the accuracy of our result we are able to estimate the kinetic energy as an error, instead of approximating it with bosonic operators. We can then carry out a bosonization procedure similar to the one of [14–16]. The resulting effective bosonic operator has continuous spectrum, but has a quite simple structure. This allows us to calculate the bottom of its spectrum without Bogoliubov diagonalizing and to find approximate ground states for which the bosonization errors are sufficiently small.

More precisely, we construct a linear map $\Phi$ from the Fock space of fictitious exact bosons to the Hilbert space of fermions and prove that it is almost isometric, up to errors which are $O(N^{-1 + \frac{1}{d}})$, but rapidly grow with the number of bosons. Likewise, $\Phi ^{*}H_N \Phi$ can be approximated by a quadratic bosonic Hamiltonian. We choose a state vector *f* in the bosonic Fock space and take $\Phi f$ for the trial state of $H_N$. Then, we calculate the energy in the limit $N \rightarrow \infty$ and only afterwards optimize over *f*.

## Statement of the Main Result

We represent the two-body potential *v* by Fourier series

8 $$\begin{aligned} v(x) = \sum _{k \in (2 \pi {\mathbb {Z}})^d} \hspace{-0.75em} {\widehat{v}}(k) \textrm{e}^{\textrm{i}k x}. \end{aligned}$$

*v* has to be real and even, so ${\widehat{v}}(k) = {\widehat{v}}(-k)$ is real.

Potentials *v* studied in this paper are bounded, so the differential operator $H_N$ in (1) is a self-adjoint operator on ${\mathcal {H}}_N = L^2_a(({\mathbb {T}}^d)^N)$. Its domain consists of all $f \in {\mathcal {H}}_N$ such that $\Delta f$ (understood in the sense of distributions) is also in ${\mathcal {H}}_N$. The spectrum of $H_N$ is discrete, bounded from below, and consists only of eigenvalues of finite multiplicity. The lowest eigenvalue of $H_N$ is called the ground state energy and denoted $E_N$.

In the absence of interactions (i.e. for $v=0$), $E_N$ is an eigenvalue of multiplicity 1 if and only if *N* is of the form

9 $$\begin{aligned} N = | \{ p \in (2 \pi \mathbb {Z})^d \, | \, |p| \le k_F \} | \end{aligned}$$

for some $k_F > 0$, called the Fermi momentum.

### Theorem 2

Let $d \ge 2$, $\alpha < 1 - \frac{2}{d}$. Suppose that the Fourier coefficients in (8) satisfy

10 $$\begin{aligned} {\widehat{v}}(0) \in \mathbb {R}, \qquad {\widehat{v}}(k) = {\widehat{v}}(-k) \ge 0 \text { for } k \ne 0, \qquad \sum _{k \in (2 \pi \mathbb {Z})^d} \hspace{-0.75em} |k| {\widehat{v}}(k) < \infty . \end{aligned}$$

Then, for *N* of the form (9) we have

11 $$\begin{aligned} E_N = \sum _{\begin{array}{c} p \in (2 \pi \mathbb {Z})^d \\ |p| \le k_F \end{array}} \hspace{-0.75em} |p|^2 + \frac{N^{2-\alpha }}{2} \int _{{\mathbb {T}}^d} v - \frac{N^{1-\alpha }}{2} v(0) + o(N^{1-\alpha - \frac{1}{d}}). \end{aligned}$$

Let us state a version of Theorem 2 valid for particles with spin. Let *q* be the number of spin states. For each spin value $\sigma \in \{ 1, \dots , q \}$ we let the number of particles of spin $\sigma$ be

12 $$\begin{aligned} N_\sigma = | \{ p \in (2 \pi \mathbb {Z})^d \, | \, |p| \le \lambda _\sigma k_F \} |, \end{aligned}$$

where $\lambda _\sigma$ is fixed and $k_F \rightarrow \infty$ is the same for all $\sigma$. The total number of particles is $N = \sum _\sigma N_{\sigma }$. We consider the Hilbert space of square-integrable functions on $({\mathbb {T}}^d)^N$ antisymmetric under permutations in $S_{N_1} \times \dots \times S_{N_q}$, on which we have the Hamiltonian

13 $$\begin{aligned} H_N = \sum _{i=1}^N (- \Delta _i) + N^{-\alpha } \sum _{1 \le i < j \le N} \hspace{-0.75em}v_{\sigma (i) \sigma (j)} (x_i-x_j). \end{aligned}$$

Here $\sigma (i)$ is the spin of the *i*th particle, i.e. $\sigma (i)=1$ if $i \in \{ 1, \dots , N_1 \}$, $\sigma (i)=2$ if $i \in \{ N_1 +1, \dots , N_1+N_2 \}$, etc. The potential is taken to be a real matrix in the spin space satisfying $v(x)= v(-x)^{\textrm{T}}$. In particular, its Fourier transform ${\widehat{v}}(k)$ takes values in the hermitian matrices. Under the assumptions

14 $$\begin{aligned} {\widehat{v}}(k) \text { is a positive matrix for all } k \ne 0, \qquad \sum _{k \in (2 \pi {\mathbb {Z}})^d} \hspace{-0.5em} |k| \Vert {\widehat{v}}(k) \Vert dk < \infty , \end{aligned}$$

a self-evident adjustment of the proof of Theorem 2 shows that $E_N = \inf {{\,\textrm{spec}\,}}H_N$ satisfies

15 $$\begin{aligned} E_N {=} \sum _{\sigma =1}^q \sum _{\begin{array}{c} p \in (2 \pi {\mathbb {Z}})^d \\ |p| {\le }\lambda _\sigma k_F \end{array}} \hspace{-0.75em} |p|^2 + \frac{1}{2} \sum _{\sigma , \sigma '=1}^q N_\sigma N_{\sigma '} \int _{{\mathbb {T}}^d} v_{\sigma \sigma '} {-} \frac{1}{2} \sum _{\sigma =1}^q N_\sigma v_{\sigma \sigma }(0) + o (N^{1 - \alpha - \frac{1}{d}}).\nonumber \\ \end{aligned}$$

To avoid cluttered notation, we present the proof for the slightly less general statement in Theorem 2.

## Proof

The *n* particle Hilbert space, $n \ge 1$, is defined to be ${\mathcal {H}}_n = L^2_a(({\mathbb {T}}^d)^n)$—square-integrable functions on $({\mathbb {T}}^d)^n$ antisymmetric with respect to permutations of the *n* coordinates. We put also ${\mathcal {H}}_0 = {\mathbb {C}}$. The direct sum ${\mathcal {H}} = \bigoplus _{n=0}^\infty {\mathcal {H}}_n$ is called the Fock space.

We let $a_p^*,a_p$ be the creation and annihilation operators of a particle with momentum *p*. These are bounded operators on ${\mathcal {H}}$, adjoint to each other, such that

16 $$\begin{aligned} a_p^* f (x_1, \dots , x_{n+1}) = \frac{1}{\sqrt{n+1}} \sum _{i=1}^{n+1} (-1)^{i-1} \textrm{e}^{\textrm{i}p x_i} f(x_1, \dots , x_{i-1}, x_{i+1}, \dots , x_{n+1})\nonumber \\ \end{aligned}$$

for $f \in {\mathcal {H}}_n$. We have the canonical anticommutation relations

17 $$\begin{aligned} a_p a_q + a_q a_p = a_p^* a_q^* + a_q^* a_p^* = 0, \qquad a_p a_q^* + a_q^* a_p = \delta _{p,q}, \end{aligned}$$

where $\delta _{p,q}$ is the Kronecker delta.

The plane wave state $\psi _0 \in {\mathcal {H}}_N$ is defined by

18 $$\begin{aligned} \psi _0(x_1, \dots , x_N) = \frac{1}{\sqrt{N!}} \det (\textrm{e}^{\textrm{i}p_i x_j})_{i,j=1}^N, \end{aligned}$$

where $p_1, \dots , p_N$ are all the $p \in (2 \pi \mathbb {Z})^d$ such that $|p| \le k_F$. It satisfies $\Vert \psi _0 \Vert =1$ and

19 $$\begin{aligned} a_p^* \psi _0 =0 \text { for } |p| \le k_F, \qquad a_p \psi _0 =0 \text { for } |p| > k_F. \end{aligned}$$

The normally ordered kinetic energy operator is defined by^1^

20 $$\begin{aligned} :\hspace{-0.1em}T\hspace{-0.1em}: \hspace{0.2em} = \hspace{-0.75em} \sum _{p \in (2 \pi \mathbb {Z})^d} \hspace{-0.75em} p^2 a_p^* a_p - \hspace{-0.75em} \sum _{\begin{array}{c} p \in (2 \pi \mathbb {Z})^d \\ |p| \le k_F \end{array}} \hspace{-0.75em} p^2 = -\hspace{-0.5em} \sum _{\begin{array}{c} p \in (2 \pi \mathbb {Z})^d \\ |p| \le k_F \end{array}} \hspace{-0.75em} p^2 a_p a_p^* + \hspace{-0.75em} \sum _{\begin{array}{c} p \in (2 \pi \mathbb {Z})^d \\ |p| > k_F \end{array}} \hspace{-0.75em} p^2 a_p^* a_p. \end{aligned}$$

By construction, $:T: \hspace{0.5em}\ge 0$ on ${\mathcal {H}}_N$.

For every $k \in (2 \pi \mathbb {Z})^d$ let

21 $$\begin{aligned} \rho _k = \hspace{-0.75em}\sum _{p \in (2 \pi \mathbb {Z})^d}\hspace{-0.75em} a_{p-k}^* a_p. \end{aligned}$$

We note that $\rho _k^* = \rho _{-k}$ and that $\rho _k$ restrict to commuting operators on each ${\mathcal {H}}_n$ with norm *n*. We have $\rho _0=n$ on ${\mathcal {H}}_n$.

Let us define

22 $$\begin{aligned} E_N^{(0)} = \sum _{\begin{array}{c} p \in (2 \pi \mathbb {Z})^d \\ |p| \le k_F \end{array}} \hspace{-0.75em} |p|^2 +\frac{N^{2-\alpha }}{2} \int _{{\mathbb {T}}^d} v - \frac{N^{1-\alpha }}{2} v(0). \end{aligned}$$

We have to prove that $E_N = E_N^{(0)} + o (N^{1 - \alpha - \frac{1}{d}})$.

With standard manipulations, one can derive

23 $$\begin{aligned} H_N - E_N^{(0)} = \hspace{0.5em}: \hspace{-0.1em}T \hspace{-0.1em}: + \hspace{0.25em} \frac{N^{- \alpha }}{2} \sum _{\begin{array}{c} k \in (2 \pi \mathbb {Z})^d \\ k \ne 0 \end{array}} \hspace{-0.75em} {\widehat{v}}(k) \rho _k^* \rho _k, \end{aligned}$$

in which restriction of the right hand side to ${\mathcal {H}}_N$ is implicitly understood. From this, we see immediately that, under assumptions of Theorem 2,

24 $$\begin{aligned} E_N - E_N^{(0)} \ge 0. \end{aligned}$$

Therefore, we need only an upper bound on $E_N - E_N^{(0)}$. One upper bound is obtained by considering the trial vector $\psi _0$.

25 $$\begin{aligned} E_N \le \langle \psi _0 | H_N \psi _0 \rangle = E_N^{(0)} + \frac{N^{- \alpha }}{2} \sum _{\begin{array}{c} k \in (2 \pi \mathbb {Z})^d \\ k \ne 0 \end{array}} \hspace{-0.75em} |C_k| {\widehat{v}}(k), \end{aligned}$$

where $|C_k|$ is the number of elements of the crescent set $C_k$:

26 $$\begin{aligned} C_k = \{ p \in (2 \pi \mathbb {Z})^d \, | \, |p| \le k_F, \, |p+k| > k_F \}. \end{aligned}$$

By Lemma 3, (25) gives $E= E_N^{(0)} + O(N^{1 - \alpha - \frac{1}{d}})$. A better trial state is needed to replace $O(N^{1 - \alpha - \frac{1}{d}})$ with $o(N^{1 - \alpha - \frac{1}{d}})$.

Next we will prove Lemma 3, which describes the growth of $|C_k|$ for large $k_F$. The right intuition for that may be gained by calculating the Lebesgue measure of the set

27 $$\begin{aligned} \{ p \in {\mathbb {R}}^d \, | \, |p| \le k_F, \, |p+k| > k_F \}. \end{aligned}$$

However, it is tricky to justify that the number of lattice points inside is well approximated by the volume. This is because (in the most important range of parameters: $k \ne 0$ of order 1 and very large $k_F$) the set (27) has the shape of a crescent with thickness comparable to the spacing between individual lattice points. Our argument gives lower and upper bounds with correct dependence on $k_F$, rather than the expected approximation with the volume. This is enough for our purposes.

### Lemma 3

There exist positive constants $c_1,c_2, c_3$, depending only on *d*, such that for all $k \in (2 \pi \mathbb {Z})^d$ and for all $k_F \ge c_3$ we have

28 $$\begin{aligned} c_1 k_F^{d-1} \min \{ |k|, k_F \} \le |C_k| \le c_2 k_F^{d-1} \min \{ |k|, k_F \}. \end{aligned}$$

### Proof

If $d=1$ (which is not needed in this paper), one can check (28) by a direct computation. The estimate is also clear for $k =0$, because then $C_k = \emptyset$. We assume that $d \ge 2$ and $k \ne 0$ (so $|k| \ge 2 \pi$).

Let $B(K) = \{p \in (2 \pi \mathbb {Z})^d \, | \, |p| \le K \}$. It is well known that

29 $$\begin{aligned} |B(K)|= (2 \pi )^{-d} |{\mathbb {S}}^{d-1}| |K|^d + o(K^{d-1}), \end{aligned}$$

where ${\mathbb {S}}^{d-1}$ is the $(d-1)$-dimensional unit sphere and $|{\mathbb {S}}^{d-1}|$ is its volume.

First we prove the upper bound on $|C_k|$. Consider the case $|k| \le k_F$. Then,

30 $$\begin{aligned} C_k \subset B(k_F) \setminus B(k_F-|k|), \qquad B(k_F-|k|) \subset B(k_F), \end{aligned}$$

and therefore we have

31 $$\begin{aligned} |C_k|&\le (2 \pi )^{-d}|{\mathbb {S}}^{d-1}| (k_F^d - (k_F-|k|)^d) + o(k_F^{d-1}) \nonumber \\&\le (2 \pi )^{-d}|{\mathbb {S}}^{d-1}| d \, k_F^{d-1} |k| + o (k_F^{d-1}). \end{aligned}$$

For large enough $k_F$ (independent of *k*), we can drop the $o (k_F^{d-1})$ error at the price of slightly increasing the constant in the first term.

In the case $|k| > k_F$ we use

32 $$\begin{aligned} |C_k| \le N \le c k_F^d. \end{aligned}$$

We remark that for the argument so far it would be enough to have error term $O(K^{d-1})$ in (29).

Next we prove the lower bound for $|k| \le k_F$. Let *G* be the group of linear isometries of $(2 \pi \mathbb {Z})^d$, $G = \textrm{GL}(d, \mathbb {Z}) \cap \textrm{O}(d)$. Elements of *G* have exactly one nonzero entry, equal $\pm 1$, in every row and every column. To see that, note that every unit vector with integer entries has only one nonzero entry, equal $\pm 1$. An equivalent description of *G* is that every element factorizes uniquely into a product of a permutation matrix and a diagonal matrix with entries $\pm 1$. Thus $|G|=d! 2^d$ (what will be important is that *G* is finite). For every $\omega \in {\mathbb {S}}^2$, there exists $g \in G$ such that $g \omega$ is in the set

33 $$\begin{aligned} D=\{ (\omega _1,\dots ,\omega _d) \in {\mathbb {S}}^{d-1} \, | \, 0 \le \omega _1 \le \omega _2 \le \dots \le \omega _d \}. \end{aligned}$$

Indeed, we can first apply a diagonal matrix with entries $\pm 1$ to make all components of $\omega$ non-negative and then apply a permutation matrix to obtain a non-decreasing tuple of numbers. Next, we claim that for any two $\omega ,\omega ' \in D$ we have^2^

34 $$\begin{aligned} \omega \cdot \omega ' \ge \frac{1}{\sqrt{d}}. \end{aligned}$$

Indeed, the map $(\omega _1,\dots ,\omega _d) \mapsto (\omega _1^2,\dots ,\omega _d^2)$ is a bijection between *D* and

35 $$\begin{aligned} S = \left\{ (t_1,\dots ,t_d) \in {\mathbb {R}}^d \, \Big | \, 0 \le t_1 \le \dots \le t_d, \ \sum _{i=1}^d t_i =1 \right\} . \end{aligned}$$

The set *S* is a simplex with vertices

36 $$\begin{aligned} (0,\dots ,1), (0, \dots , \tfrac{1}{2}, \tfrac{1}{2}), \dots , (\tfrac{1}{d},\dots ,\tfrac{1}{d}). \end{aligned}$$

Now parametrizing *D* in terms of corresponding points in *S*, we have

37 $$\begin{aligned} \omega \cdot \omega ' = \sum _{i=1}^d \sqrt{t_i\phantom {t_i'}} \sqrt{t_i'} \qquad (t_i = \omega _i^2, \, t_i' = \omega _i'^2). \end{aligned}$$

For fixed $t=(t_1,\dots ,t_d) \in S$, this is a concave function of $t' = (t_1',\dots ,t_d')\in S$, and analogously for the dependence on $t'$ for fixed *t*. It follows that the minimum of $\omega \cdot \omega '$ over all $\omega , \omega ' \in D$ is attained at some $\omega , \omega '$ corresponding to $t,t'$ being vertices of *S* (a continuous concave function defined on a compact convex set attains its minimum on one of the extreme points of the set). It is easy to verify that the vertices corresponding to the minimal value are the first and the last one in (36), i.e.

38 $$\begin{aligned} \omega = \left( \phantom {\frac{1}{\sqrt{d}}} 0,\dots ,1 \right) , \quad \omega ' = \left( \frac{1}{\sqrt{d}},\dots ,\frac{1}{\sqrt{d}} \right) \end{aligned}$$

This completes the proof of (34). The important consequence is that for any two $\omega , \omega ' \in {\mathbb {S}}^d$ there exists $g \in G$ such that $\omega \cdot g\omega ' \ge \frac{1}{\sqrt{d}}$. Indeed, we can find $g_1,g_2 \in G$ such that $g_1 \omega \in D, g_2 \omega ' \in D$. Then

39 $$\begin{aligned} \frac{1}{\sqrt{d}} \le g_1 \omega \cdot g_2 \omega ' = \omega \cdot g_1^{-1} g_2 \omega ', \end{aligned}$$

so we can take $g = g_1^{-1}g_2$.

Let $p \in B(k_F) \setminus B\Big (k_F - \frac{1}{\sqrt{d}}|k| \Big )$. Choose $g \in G$ such that $p \cdot gk \ge \frac{1}{\sqrt{d}} |p| |k|$. Then,

40 $$\begin{aligned} |p + gk|^2&\ge |p|^2 + \frac{2}{\sqrt{d}} |p| |k| + |k|^2> \Big (k_F- \frac{1}{\sqrt{d}} |k| \Big )^2 + \frac{2|k|}{\sqrt{d}} \Big (k_F - \frac{1}{\sqrt{d}} |k| \Big ) + |k|^2 \nonumber \\&= k_F^2 + \frac{d-1}{d} |k|^2 > k_F^2, \end{aligned}$$

so $p \in C_{g k}$. We proved that

41 $$\begin{aligned} B(k_F) \setminus B \Big ( k_F - \frac{1}{\sqrt{d}} |k| \Big ) \subset \bigcup _{g \in G} C_{g k}. \end{aligned}$$

Since $|C_{gk}|= |C_k|$, we have

42 $$\begin{aligned} |C_k| \ge \frac{1}{|G|} \left| \bigcup _{g \in G} C_{g k} \right| \ge \frac{1}{|G|} \left| B(k_F) \setminus B \Big ( k_F - \frac{1}{\sqrt{d}} |k| \Big ) \right| . \end{aligned}$$

Next, we have

43 $$\begin{aligned} \left| B(k_F) \setminus B \Big ( k_F - \frac{1}{\sqrt{d}} |k| \Big ) \right|&= (2 \pi )^{-d} |{\mathbb {S}}^{d-1}| \Big ( k_F^d - \Big ( k_F - \frac{1}{\sqrt{d}} |k| \Big )^d \Big ) + o(k_F^{d-1}) \nonumber \\&\ge (2 \pi )^{-d} |{\mathbb {S}}^{d-1}| d \, \Big ( k_F- \frac{|k|}{\sqrt{d}} \Big )^{d-1} \frac{|k|}{\sqrt{d}} +o(k_F^{d-1}) . \end{aligned}$$

Again restricting to large enough $k_F$, this may be lower bounded by $c k_F^{d-1} |k|$. Here we are using that the error term in (29) is $o(k_F^{d-1})$ and not just $O(k_F^{d-1})$, in which case there could in principle be cancelations between the main term and the error term in (43).

If $|k| > k_F$, we have $C_k \cup C_{-k} = B(k_F)$, and hence

44 $$\begin{aligned} |C_k| \ge \frac{1}{2} N \ge c k_F^{d}. \end{aligned}$$

$$\square $$

In what follows, we take $k_F$ large enough that (28) holds.

It is useful to split $\rho _k$ with $k \ne 0$ as follows:

45 $$\begin{aligned} \rho _k = b_k + b_{-k}^* + d_k, \end{aligned}$$

where we introduce (all *p* here and in other sums below are in $(2 \pi \mathbb {Z})^d$):

46 $$\begin{aligned} b_{-k}^*&= \hspace{-0.75em} \sum _{\begin{array}{c} |p| \le k_F \\ |p-k| > k_F \end{array}} \hspace{-0.75em} a_{p-k}^* a_p, \end{aligned}$$

47 $$\begin{aligned} b_k&= \hspace{-0.75em} \sum _{\begin{array}{c} |p| > k_F \\ |p-k| \le k_F \end{array}} \hspace{-0.75em} a_{p-k}^* a_{p}, \end{aligned}$$

48 $$\begin{aligned} d_k&= -\hspace{-1.25em} \sum _{|p|, |p-k| \le k_F } \hspace{-1.5em} a_p a_{p-k}^*+ \hspace{-1.25em} \sum _{|p|, |p-k| > k_F } \hspace{-1.5em} a_{p-k}^* a_p . \end{aligned}$$

The operator $b_{-k}^*$ is indexed by $-k$ so that $b_k$ and $b_k^*$ are adjoint to each other.

We have the commutation rules

49 $$\begin{aligned} {[}b_k,b_q]=[b_k^*,b_q^*]= 0, \nonumber \\ {[}b_k, b_q^*] = |C_k| \delta _{k,q} +:\hspace{-0.25em} [b_k,b_q^*] \hspace{-0.25em}:, \end{aligned}$$

where we introduced

50 $$\begin{aligned} :\hspace{-0.25em}[b_k,b_q^*]\hspace{-0.25em}: \hspace{0.25em}= -\hspace{-2em} \sum _{\begin{array}{c} |p|, |p+q-k| \le k_F \\ |p+q|> k_F \end{array}} \hspace{-1.75em} a_p a_{p+q-k}^* - \hspace{-2em} \sum _{\begin{array}{c} |p-k| \le k_F \\ |p+q-k|, |p| > k_F \end{array}} \hspace{-1.75em} a_{p+q-k}^* a_{p}. \end{aligned}$$

We will not use it, but we remark that $-:[b_k,b_k^*]: \hspace{0.25em} \ge 0$.

The basis of the bosonic approximation is that $d_k$ and $:[b_k,b_q^*]:$ annihilate $\psi _0$, and they are small when acting on states close to $\psi _0$. The closeness is measured by the number of excitations operator

51 $$\begin{aligned} {\mathcal {N}} = \frac{1}{2} \sum _{|p| \le k_F} \hspace{-0.25em} a_p a_p^* + \frac{1}{2} \sum _{|p| > k_F} \hspace{-0.25em} a_p^* a_p, \end{aligned}$$

see Lemma 4. We remark that the two terms in (51) are equal on ${\mathcal {H}}_N$. The operator ${\mathcal {N}}$ is self-adjoint with spectrum ${\mathbb {N}}$.

The bounds in Lemma 4 are known, see e.g. [14, Lemmas 4.1, 4.2]. For convenience of the reader, we include the proof using our notation.

### Lemma 4

Let $$\psi \in {\mathcal {H}}$$.

1. If $$\Vert {\mathcal {N}}^{\frac{1}{2}} \psi \Vert < \infty $$, then $$\Vert b_k \psi \Vert \le |C_k|^{\frac{1}{2}} \Vert {\mathcal {N}}^{\frac{1}{2}} \psi \Vert $$, $$\Vert b_k^* \psi \Vert \le |C_k|^{\frac{1}{2}} \Vert ({\mathcal {N}} + 1)^{\frac{1}{2}} \psi \Vert $$.
2. If $\Vert {\mathcal {N}} \psi \Vert < \infty$, then $\Vert \hspace{-0.25em}: \hspace{-0.25em} [b_k,b_q^*] \hspace{-0.25em}: \hspace{-0.25em}\psi \Vert \le 2 \Vert {\mathcal {N}} \psi \Vert$ and $\Vert d_k \psi \Vert \le 2 \Vert {\mathcal {N}} \psi \Vert$.

### Proof

By the triangle inequality and Cauchy–Schwarz:

52 $$\begin{aligned} \Vert b_k \psi \Vert = \Big \Vert \hspace{-0.75em} \sum _{ \begin{array}{c} |p|> k_F \\ |p-k| \le k_F \end{array}} \hspace{-1em} a_{p-k}^* a_p \psi \Big \Vert&\le \hspace{-0.75em} \sum _{ \begin{array}{c} |p|> k_F \\ |p-k| \le k_F \end{array}} \hspace{-1em} \Vert a_{p-k}^* a_p \psi \Vert \nonumber \\&\le |C_k|^{\frac{1}{2}} \Big ( \hspace{-0.5em} \sum _{ \begin{array}{c} |p| > k_F \\ |p-k| \le k_F \end{array}} \hspace{-1em} \Vert a_{p-k}^* a_p \psi \Vert ^2 \Big )^{\frac{1}{2}}. \end{aligned}$$

Now note that $a_{p-k}^*$ and $a_p$ anticommute because we consider $b_k$ only for $k \ne 0$ (its definition (47) gives $b_k=0$ if we use it for $k=0$, so the bound claimed in the lemma would be trivially true anyway). Therefore,

53 $$\begin{aligned} \Vert a_{p-k}^* a_p \psi \Vert ^2&= \langle a_p^* a_p \psi | a_{p-k} a_{p-k}^* \psi \rangle \le \Vert a_p^* a_p \psi \Vert \Vert a_{p-k} a_{p-k}^* \psi \Vert = \Vert a_p \psi \Vert \Vert a_{p-k}^* \psi \Vert \nonumber \\&\le \frac{1}{2} \Vert a_p \psi \Vert ^2 + \frac{1}{2} \Vert a_{p-k}^* \psi \Vert ^2 = \langle \psi | \tfrac{1}{2} (a_p^* a_p + a_{p-k} a_{p-k}^*) \psi \rangle , \end{aligned}$$

where we used Cauchy-Schwarz. Next, we use this result in (52):

54 $$\begin{aligned} \Vert b_k \psi \Vert&\le |C_k|^{\frac{1}{2}} \Big ( \hspace{-0.5em} \sum _{ \begin{array}{c} |p|> k_F \\ |p-k| \le k_F \end{array}} \hspace{-1em} \langle \psi | \tfrac{1}{2} (a_p^* a_p + a_{p-k} a_{p-k}^* ) \psi \rangle \Big )^{\frac{1}{2}} \nonumber \\&\le |C_k|^{\frac{1}{2}} \langle \psi | \Big ( \tfrac{1}{2} \hspace{-0.5em} \sum _{|p| > k_F} \hspace{-0.5em} a_p^* a_p + \tfrac{1}{2} \hspace{-1em} \sum _{|p-k| \le k_F} \hspace{-0.5em} a_{p-k} a_{p-k}^* \Big ) \psi \rangle ^{\frac{1}{2}} \nonumber \\&= |C_k|^{\frac{1}{2}} \langle \psi | {\mathcal {N}} \psi \rangle ^{\frac{1}{2}} = |C_k|^{\frac{1}{2}} \Vert {\mathcal {N}}^{\frac{1}{2}} \psi \Vert . \end{aligned}$$

The inequality in the second line of (54) is true because we have relaxed the condition on elements *p* over which the summation is performed, and each term is non-negative. This completes the proof of the first inequality in 1. Next, we note that for any vector $\phi \in {\mathcal {H}}$ we have the orthogonal decomposition

55 $$\begin{aligned} \phi = \sum _{n=0}^\infty \phi _n, \qquad {\mathcal {N}} \phi _n = n \phi _n. \end{aligned}$$

Moreover, an application of $b_k$ lowers ${\mathcal {N}}$ by 1, i.e. $(b_k \phi )_n = b_k \phi _{n+1}$. Thus

56 $$\begin{aligned} | \langle \phi | b_k^* \psi \rangle | =| \langle b_k \phi | \psi \rangle | = \left| \sum _{n=0}^\infty \langle b_k \phi _{n+1} | \psi _n \rangle \right| \le \sum _{n=0}^\infty \Vert b_k \phi _{n+1} \Vert \Vert \psi _n \Vert . \end{aligned}$$

The inequality established in (54) applied to $\phi _{n+1}$ in place of $\psi$ now gives:

57 $$\begin{aligned} | \langle \phi | b_k^* \psi \rangle |&\le \sum _{n=0}^\infty |C_k|^{\frac{1}{2}} (n+1)^{\frac{1}{2}} \Vert \phi _{n+1} \Vert \Vert \psi _n \Vert \nonumber \\&\le |C_k|^{\frac{1}{2}} \Vert \phi \Vert \left( \sum _{n=0}^\infty (n+1) \Vert \psi _n \Vert ^2 \right) ^{\frac{1}{2}} = |C_k|^{\frac{1}{2}} \Vert \phi \Vert \Vert ({\mathcal {N}}+1)^{\frac{1}{2}} \psi \Vert , \end{aligned}$$

where the inequality in the second line follows from Cauchy–Schwarz. This completes the proof of 1.

2. We present the proof of the bound for $d_k$. The proof for $: \hspace{-0.25em} [b_k,b_q^*] \hspace{-0.25em}:$ is entirely analogous. The operator $d_k$ preserves the eigenspaces of ${\mathcal {N}}$, so it is enough to show that the inequality

58 $$\begin{aligned} | \langle \phi | d_k \psi \rangle | \le 2 n \Vert \phi \Vert \Vert \psi \Vert \end{aligned}$$

holds under the assumption that ${\mathcal {N}} \phi = n \phi$ and ${\mathcal {N}} \psi = n \psi$. We have

59 $$\begin{aligned} | \langle \phi | d_k \psi \rangle |&= \Big | - \hspace{-1.25em}\sum _{|p|,|p-k| \le k_F } \hspace{-1.25em}\langle a_p^* \phi | a_{p-k}^* \psi \rangle +\hspace{-1.25em} \sum _{|p|,|p-k|>k_F }\hspace{-1.25em} \langle a_{p-k} \phi | a_p \psi \rangle \Big | \nonumber \\&\le \hspace{-1.25em} \sum _{|p|,|p-k| \le k_F } \hspace{-1.25em} \Vert a_p^* \phi \Vert \Vert a^*_{p-k} \psi \Vert + \hspace{-1.25em}\sum _{|p|,|p-k|>k_F} \hspace{-1.25em} \Vert a_{p-k} \phi \Vert \Vert a_p \psi \Vert \nonumber \\&\le \Big ( \hspace{-0.5em} \sum _{|p| \le k_F} \hspace{-0.5em} \Vert a_p^* \phi \Vert ^2 + \hspace{-0.75em} \sum _{|p-k|> k_F} \hspace{-1em} \Vert a_{p-k} \phi \Vert ^2 \Big )^{\frac{1}{2}} \Big ( \hspace{-0.75em} \sum _{|p-k| \le k_F} \hspace{-1em} \Vert a_{p-k}^* \psi \Vert ^2 + \hspace{-0.5em} \sum _{|p| > k_F} \hspace{-0.5em} \Vert a_{p} \psi \Vert ^2 \Big )^{\frac{1}{2}}, \end{aligned}$$

where in the first inequality we apply Cauchy–Schwarz and in the second inequality we apply Cauchy-Schwarz and then relax some summation conditions. The expression obtained in (59) may be rewritten as

60 $$\begin{aligned} \langle \phi | 2 {\mathcal {N}} \phi \rangle ^{\frac{1}{2}} \langle \psi | 2 {\mathcal {N}} \psi \rangle ^{\frac{1}{2}} = 2n \Vert \phi \Vert \Vert \psi \Vert . \end{aligned}$$

$$\square $$

It is convenient to introduce normalized approximately bosonic operators

61 $$\begin{aligned} \varphi _k = |C_k|^{-\frac{1}{2}} b_k, \quad \varphi _k^* = |C_k|^{-\frac{1}{2}} b_k^*, \quad : \hspace{-0.25em} [\varphi _k,\varphi _q^*] \hspace{-0.25em}: = |C_k|^{-\frac{1}{2}} |C_q|^{-\frac{1}{2}}: \hspace{-0.25em} [b_k,b_q^*] \hspace{-0.25em}:.\nonumber \\ \end{aligned}$$

By Lemma 3, this is a correct definition because $|C_k| \ne 0$ for all *k* if $k_F$ is large enough.

Informally, what follows is based on the approximation

62 $$\begin{aligned} \rho _k \approx |C_k|^{\frac{1}{2}} (\varphi _k + \varphi _{-k}^*), \qquad [\varphi _k,\varphi _q^*] \approx \delta _{k,q}. \end{aligned}$$

Let ${\mathcal {F}}$ be the bosonic Fock space over $\ell ^2((2 \pi \mathbb {Z})^d \setminus \{ 0 \})$. We denote the vacuum vector of ${\mathcal {F}}$ by $\Omega$ and the creation and annihilation operators corresponding to elements of $(2 \pi \mathbb {Z})^d \setminus \{ 0 \}$ by $e_k^*, e_k$. Most of the time we will work in the dense subspace ${\mathcal {D}} \subset {\mathcal {F}}$ of vectors obtained from $\Omega$ by acting with a polynomial in the operators $e_k^*$.

If $S \subset (2 \pi \mathbb {Z})^d \setminus \{ 0 \}$ is finite, we let ${\mathcal {D}}^S \subset {\mathcal {D}}$ be the subspace obtained by acting on $\Omega$ with polynomials in $e_k^*$ with the restriction $k \in S$. By definition of ${\mathcal {D}}$, every element of ${\mathcal {D}}$ is in some ${\mathcal {D}}^S$:

63 $$\begin{aligned} {\mathcal {D}} = \bigcup _{S} {\mathcal {D}}^S. \end{aligned}$$

For a natural number *m*, we let ${\mathcal {D}}_{m} \subset {\mathcal {D}}$ be the *m* particle subspace, i.e. the linear span of vectors $e_{k_1}^* \cdots e_{k_m}^* \Omega$. We introduce also

64 $$\begin{aligned} {\mathcal {D}}_{\le m} = \bigoplus _{i=0}^m {\mathcal {D}}_i, \qquad {\mathcal {D}}^S_{ m} = {\mathcal {D}}^S \cap {\mathcal {D}}_{ m}, \qquad {\mathcal {D}}^S_{\le m} = {\mathcal {D}}^S \cap {\mathcal {D}}_{\le m}. \end{aligned}$$

We note that

65 $$\begin{aligned} \dim ({\mathcal {D}}_{\le m}^S) {=} \left( {\begin{array}{c}m + |S|\\ m\end{array}}\right) , \quad \dim ({\mathcal {D}}_{ m}^S) {=} \left( {\begin{array}{c}m + |S|-1\\ m\end{array}}\right) , \quad {\mathcal {D}} {=} \bigcup _{m, S} {\mathcal {D}}^S_{\le m}.\nonumber \\ \end{aligned}$$

We define a linear map $\Phi : {\mathcal {D}} \rightarrow L^2_{\textrm{a}}({\mathbb {T}}^{dN})$ by

66 $$\begin{aligned} \Phi (e_{k_1}^* \cdots e_{k_m}^* \Omega ) = \varphi _{k_1}^* \cdots \varphi _{k_m}^* \psi _0. \end{aligned}$$

Equivalently, $\Phi$ is uniquely determined by the conditions

67 $$\begin{aligned} \Phi (\Omega )=\psi _0, \qquad \Phi e_k^* = \varphi _k^* \Phi . \end{aligned}$$

We note that $\Phi$ depends on $k_F$.

We let $\Phi ^S_m$ and $\Phi ^S_{\le m}$ be the restrictions of $\Phi$ to ${\mathcal {D}}^S_m$ and ${\mathcal {D}}^S_{\le m}$, respectively. These are linear operators on finite-dimensional Hilbert spaces, so they are bounded.

Let ${\mathcal {D}}^*$ be the space of antilinear functionals on ${\mathcal {D}}$ (not necessarily continuous). We have a canonical embedding ${\mathcal {D}} \rightarrow {\mathcal {D}}^*$ given by $f \mapsto \langle \cdot | f \rangle$. If $f \in {\mathcal {D}}^*$ and $g \in {\mathcal {D}}$, we denote the evaluation of *f* at *g* by $\langle g|f \rangle$ and put $\langle f|g \rangle := \overline{\langle g|f \rangle }$.

We define the adjoint of $\Phi$ to be the map

68 $$\begin{aligned} \Phi ^*: L^2_{\textrm{a}} ({\mathbb {T}}^{dN}) \rightarrow {\mathcal {D}}^*, \qquad \langle g | \Phi ^* f \rangle = \langle \Phi g | f \rangle \text { for } g \in {\mathcal {D}}, \ f \in L^2_{\textrm{a}}({\mathbb {T}}^{dN}). \end{aligned}$$

Our $\Phi ^*$ is an extension of the standard operator adjoint. It is defined on all of $L^2_{\textrm{a}} ({\mathbb {T}}^{dN})$, at the price of being valued in the (very large) space ${\mathcal {D}}^*$. We expect that working with ${\mathcal {D}}^*$ is not strictly necessary, but it is convenient because it allows us to not worry about domain issues in the (essentially algebraic) calculations below.

Note that if $g \in {\mathcal {D}}^S_{\le m}$, then

69 $$\begin{aligned} \langle g | \Phi ^* f \rangle = \langle g | (\Phi ^S_{\le m})^* f \rangle , \end{aligned}$$

and $(\Phi ^S_{\le m})^*$ is a bounded operator $L^2_{\textrm{a}}({\mathbb {T}}^{dN}) \rightarrow {\mathcal {D}}^{S}_{\le m}$.

### Lemma 5

For any $k_1, \dots , k_m \in (2 \pi {\mathbb {Z}})^d \setminus \{ 0 \}$ and $q_1, \dots , q_m \in (2 \pi {\mathbb {Z}})^d \setminus \{ 0 \}$ (not necessarily pairwise distinct) we have the bound

70 $$\begin{aligned} |\langle e^*_{k_m} \cdots e_{k_1}^*\Omega | (\Phi ^* \Phi -1) e^*_{q_m} \cdots e^*_{q_1} \Omega \rangle | \le c \left( {\begin{array}{c}m\\ 2\end{array}}\right) m! k_F^{1-d}, \end{aligned}$$

where *c* is a universal constant. In particular, we have

71 $$\begin{aligned} \Vert (\Phi ^{S}_{\le m})^* \Phi _{\le m}^S-1 \Vert \le c \left( {\begin{array}{c}m+|S|-1\\ m\end{array}}\right) \left( {\begin{array}{c}m\\ 2\end{array}}\right) m! k_F^{1-d}. \end{aligned}$$

Hence (assuming $d \ge 2$) for any $f \in {\mathcal {D}}$ we have

72 $$\begin{aligned} \lim _{k_F \rightarrow \infty } \Vert \Phi f \Vert = \Vert f \Vert . \end{aligned}$$

### Proof

For every $m \ge 0$, we define

73 $$\begin{aligned} \epsilon _m(k_m, \dots , k_1 ; q_m , \dots , q_1) =&\langle e^*_{k_m} \cdots e^*_{k_1} \Omega | (\Phi ^* \Phi -1) e^*_{q_m} \cdots e_{q_1}^* \Omega \rangle . \end{aligned}$$

The goal is to show

74 $$\begin{aligned} |\epsilon _m(k_m, \dots , k_1; q_m, \dots , q_1)| \le c \left( {\begin{array}{c}m\\ 2\end{array}}\right) m! k_F^{1-d}, \end{aligned}$$

with a constant *c* described later in the proof.

We proceed by induction on *m*. The cases $m=0$ and $m=1$ are trivial. Using the Leibniz rule for commutators and decomposition (49), we derive

75 $$\begin{aligned} \langle&e^*_{k_{m+1}} \cdots e^*_{k_1} \Omega |\Phi ^* \Phi e^*_{q_{m+1}} \cdots e^*_{q_1} \Omega \rangle = \langle \varphi _{k_{m+1}} ^* \cdots \varphi _{k_1}^* \psi _0 | \varphi _{q_{m+1}}^* \cdots \varphi _{q_1}^* \psi _0 \rangle \nonumber \\&= \sum _{i=1}^{m+1} \langle \varphi _{k_m}^* \cdots \varphi _{k_1}^* \psi _0 | \varphi _{q_{m+1}}^* \cdots [\varphi _{k_{m+1}}, \varphi _{q_i}^*] \cdots \varphi _{q_1}^* \psi _0 \rangle \nonumber \\&= \sum _{i=1}^{m+1} \Bigg [ \delta _{q_i}^{k_{m+1}} \langle e_{k_m}^* \cdots e^*_{k_1} \Omega | \Phi ^* \Phi e_{q_{m+1}}^* \cdots e_{q_{i+1}}^* e_{q_{i-1}}^* \cdots e_{q_1}^* \Omega \rangle \nonumber \\&\quad + \langle \varphi _{k_m}^* \cdots \varphi _{k_1}^* \psi _0 | \varphi _{q_{m+1}}^* \cdots : \hspace{-0.25em} [\varphi _{k_{m+1}}, \varphi _{q_i}^*] \hspace{-0.25em} : \cdots \varphi _{q_1}^* \psi _0 \rangle \Bigg ]. \end{aligned}$$

In the first term of the summand we use $\Phi ^* \Phi = 1 + (\Phi ^* \Phi -1)$ and the identity

76 $$\begin{aligned}&\sum _{i=1}^{m+1} \delta _{q_i}^{k_{m+1}}\langle e_{k_m}^* \cdots e^*_{k_1} \Omega | e_{q_{m+1}}^* \cdots e_{q_{i+1}}^* e_{q_{i-1}}^* \cdots e_{q_1}^* \Omega \rangle \nonumber \\&\quad = \langle e_{k_{m+1}}^* \cdots e^*_{k_1} \Omega | e_{q_{m+1}}^* \cdots e_{q_1}^* \Omega \rangle . \end{aligned}$$

This allows one to rewrite (75) in the form77By the induction hypothesis (74),

78 $$\begin{aligned} |\epsilon _{m+1}(k_{m+1}, \dots , k_1 ; q_{m+1}, \dots , q_1) | \le (m+1) c \left( {\begin{array}{c}m\\ 2\end{array}}\right) m! k_F^{1-d} \quad \nonumber \\ \qquad + \sum _{i=1}^{m+1} \Vert \varphi _{k_m}^* \cdots \varphi _{k_1}^* \psi _0 \Vert \Vert \varphi _{q_{m+1}}^* \cdots : \hspace{-0.25em} [\varphi _{k_{m+1}},\varphi _{q_i}^*] \hspace{-0.25em} : \cdots \varphi _{q_1}^* \psi _0 \Vert . \end{aligned}$$

By 1. in Lemma 4 we have

79 $$\begin{aligned} \Vert \varphi _{k_m}^* \cdots \varphi _{k_1}^* \psi _0 \Vert \le m!^{\frac{1}{2}} \Vert \varphi _{k_{m-1}}^* \cdots \varphi _{k_1}^* \psi _0 \Vert \le \cdots \le m!^{\frac{1}{2}}, \end{aligned}$$

where the dots indicate repeating the same step additional $m-1$ times. Similarly, using 1. and 2. in Lemma 4 we obtain

80 $$\begin{aligned} \Vert \varphi _{q_{m+1}}^* \cdots :\hspace{-0.25em} [\varphi _{k_{m+1}},\varphi _{q_i}^*] \hspace{-0.25em}: \cdots \varphi _{q_1}^* \psi _0 \Vert \le \frac{2 m!^{\frac{1}{2}}(i-1)}{|C_{k_{m+1}}|^{\frac{1}{2}}|C_{q_i}|^{\frac{1}{2}}}. \end{aligned}$$

This and Lemma 3 imply that there exists a constant $c' > 0$ such that

81 $$\begin{aligned} \Vert \varphi _{q_{m+1}}^* \cdots :\hspace{-0.25em} [\varphi _{k_{m+1}},\varphi _{q_i}^*] \hspace{-0.25em}: \cdots \varphi _{q_1}^* \psi _0 \Vert \le 2c' k_F^{1-d} m!^{\frac{1}{2}} (i-1). \end{aligned}$$

Next, we use (79) and (81) in (78) to obtain

82 $$\begin{aligned} |\epsilon _{m+1}(k_{m+1}, \dots , k_1; q_{m+1}, \dots , q_1) | \le (m+1)! k_F^{1-d} \Big ( c \frac{m(m-1)}{2}+ c'm \Big ).\nonumber \\ \end{aligned}$$

We see that if we choose *c* to be equal $c'$, then the parenthesis in (82) equals $$c \left( {\begin{array}{c}m+1\\ 2\end{array}}\right) $$, so we get (74) for $m+1$.

The Hilbert space ${\mathcal {D}}^S_m$ has an orthonormal basis consisting of vectors

83 $$\begin{aligned} \frac{1}{\sqrt{m_1! \cdots m_t!}} (e_{k_1}^*)^{m_1} \cdot (e_{k_m}^*)^{m_t} \Omega , \end{aligned}$$

where $k_1,\dots ,k_t$ is an enumeration of all the elements of *S* and the indices $m_i \in {\mathbb {N}}$ satisfy $m_1 + \dots + m_t =m$. The bound (70) constrains the matrix elements of $(\Phi ^S_m)^* \Phi ^S_m-1$ relative to this basis. Recalling that for an $n \times n$ matrix *T* we have $\Vert T \Vert \le n \max _{i,j} |T_{ij}|$, we obtain the bound

84 $$\begin{aligned} \Vert (\Phi ^S_m)^* \Phi ^S_m-1 \Vert&\le \dim ({\mathcal {D}}^S_m) \max _{m_1 + \dots + m_t =m} \frac{1}{m_1! \cdots m_t!} c \left( {\begin{array}{c}m\\ 2\end{array}}\right) m! k_F^{1-d} \nonumber \\&\le c \left( {\begin{array}{c}m+|S|-1\\ m\end{array}}\right) \left( {\begin{array}{c}m\\ 2\end{array}}\right) m! k_F^{1-d} . \end{aligned}$$

The orthogonal decomposition ${\mathcal {D}}^S_{\le m} = \bigoplus _{i=0}^m {\mathcal {D}}^S_{i}$ is preserved by the operator $(\Phi ^{S}_{\le m})^* \Phi _{\le m}^S-1$, so

85 $$\begin{aligned} \Vert (\Phi ^{S}_{\le m})^* \Phi _{\le m}^S -1 \Vert&\le \max _{0 \le n \le m} \Vert (\Phi ^{S}_{n})^* \Phi _{n}^S -1 \Vert \nonumber \\&\le \max _{0 \le n \le m} c \left( {\begin{array}{c}n+|S|-1\\ n\end{array}}\right) \left( {\begin{array}{c}n\\ 2\end{array}}\right) n! k_F^{1-d} \nonumber \\&= c \left( {\begin{array}{c}m+|S|-1\\ m\end{array}}\right) \left( {\begin{array}{c}m\\ 2\end{array}}\right) m! k_F^{1-d} . \end{aligned}$$

$$\square $$

Let us split $H_N= E_N^{(0)} +H^{(1)}+H^{(2)}$, where

86 $$\begin{aligned} H^{(1)} =\,&\frac{N^{-\alpha }}{2} \sum _{k \ne 0} |C_k| {\widehat{v}}(k) (\varphi _k^* + \varphi _{-k})(\varphi _{-k}^* + \varphi _k) , \nonumber \\ H^{(2)} =\,&: \hspace{-0.2em}T \hspace{-0.2em}: + \frac{N^{-\alpha }}{2} \sum _{k \ne 0} {\widehat{v}}(k) \left( (b_k^* + b_{-k}) d_k + d_{-k} (b_{-k}^* +b_k) + d_{-k}d_k \right) . \end{aligned}$$

Furthermore, let us define the self-adjoint operators

87 $$\begin{aligned} H^B&= \frac{N^{-\alpha }}{2} \sum _{k \ne 0} |C_k| {\widehat{v}}(k) (e_k^*+e_{-k})(e_{-k}^* + e_k), \nonumber \\ {\widetilde{H}}^B&= \sum _{k \ne 0} |k| {\widehat{v}}(k) (e_k^*+e_{-k})(e_{-k}^* + e_k). \end{aligned}$$

We will treat $$H^{(2)}$$ as an error term and $$H^B$$ as an approximation to $$ H^{(1)}$$. The operator $${\widetilde{H}}^B$$ does not depend on $$k_F$$, and by Lemma 3 we have

88 $$\begin{aligned} 0 \le H^B \le c N^{1- \alpha - \frac{1}{d}} {\widetilde{H}}^B \end{aligned}$$

for some $c >0$. The bottom of the spectrum of ${\widetilde{H}}^B$ is 0, as we show in Lemma 6.

### Lemma 6

We have

89 $$\begin{aligned} \inf _{\begin{array}{c} f \in {\mathcal {D}} \\ \Vert f \Vert =1 \end{array}} \langle f | {\widetilde{H}}^B f \rangle = 0. \end{aligned}$$

### Proof

If $f \in {\mathcal {D}}$, then we have $\langle f | {\widetilde{H}}^B f \rangle \ge 0$. We have to prove that this matrix element can be made as small as desired by choosing a suitable *f* with $\Vert f \Vert =1$. Let $S \subset (2 \pi {\mathbb {Z}})^d \setminus \{ 0 \}$ be a finite set such that if $k \in S$, then $-k \not \in S$. Let $c_0,c_1,\dots$ be a sequence of complex numbers with only finitely many nonzero entries. We consider the vector

90 $$\begin{aligned} f = \prod _{k \in S} \left( \sum _{n=0}^M c_n \frac{(e_k^*e_{-k}^*)^n}{n!} \right) \Omega \in {\mathcal {D}}. \end{aligned}$$

Using the canonical commutation rules one can verify that vectors $\prod _{k \in S} \frac{(e_k^* e_{-k})^n}{n!}$ form an orthonormal set in ${\mathcal {F}}$, and therefore

91 $$\begin{aligned} \Vert f \Vert ^2 = \prod _{k \in S} \left( \sum _{n=0}^\infty |c_n|^2 \right) . \end{aligned}$$

We will achieve the condition $\Vert f \Vert =1$ by choosing the coefficients $c_n$ satisfying

92 $$\begin{aligned} \sum _{n=0}^\infty |c_n|^2 =1. \end{aligned}$$

Another simple calculation using canonical commutation relations shows that

93 $$\begin{aligned} \langle f | (e_k^* + e_{-k} ) (e_{-k} + e_k) f \rangle = {\left\{ \begin{array}{ll} \epsilon , &  k \in S \cup -S, \\ 1, &  \text {otherwise}, \end{array}\right. } \nonumber \\ \text {where: } \epsilon := \sum _{n=0}^\infty (n+1) \overline{c_{n+1}} c_n + \sum _{n=1}^\infty n \overline{c_{n-1}} c_n + \sum _{n=0}^\infty (2n+1) |c_n|^2. \end{aligned}$$

It follows that

94 $$\begin{aligned} \langle f | {\widetilde{H}}^B f \rangle = \epsilon \hspace{-0.75em} \sum _{k \in S \cup -S} \hspace{-0.75em} |k| {\widehat{v}}(k) + \hspace{-1.5em} \sum _{\begin{array}{c} k \in (2 \pi {\mathbb {Z}})^d \setminus \{ 0 \} \\ k \not \in S \cup -S \end{array}} \hspace{-1.5em} |k| {\widehat{v}}(k). \end{aligned}$$

Below we will demonstrate that we can choose the coefficients $c_n$ so that $\epsilon$ becomes as small as desired. The convergence of the series $\sum _{k} |k| {\widehat{v}}(k)$ then implies that the right hand side of (94) can be made arbitrarily small by taking a small $\epsilon$ and a large set *S*.

We choose a positive integer *M* and let

95 $$\begin{aligned} c_n = {\left\{ \begin{array}{ll}\frac{1}{Z^{\frac{1}{2}}} (-1)^n \left( 1 - \frac{n}{M} \right) , &  n \le M \\ 0, &  n > M. \end{array}\right. } \end{aligned}$$

We take *Z* to be a positive number chosen so that (92) holds, i.e.

96 $$\begin{aligned} Z = \sum _{n=1}^M \left( 1 - \frac{n}{M} \right) ^2 = \frac{(M+1)(2M+1)}{6M}. \end{aligned}$$

Evaluating $\epsilon$ we find

97 $$\begin{aligned} \epsilon = \frac{3}{2M+1}. \end{aligned}$$

Now take *M* very large. $\square$

### Lemma 7

We have a bound

98 $$\begin{aligned} \Vert (\varphi _k \Phi -\Phi e_k)e^*_{q_m} \cdots e^*_{q_1} \Omega \Vert \le c k_F^{1-d} \sqrt{(m-1)!} \left( {\begin{array}{c}m\\ 2\end{array}}\right) , \end{aligned}$$

and therefore

99 $$\begin{aligned} \Vert \varphi _k \Phi _{\le m}^S - \Phi ^S_{\le m} e_k \Vert \le c k_F^{1-d} \left( {\begin{array}{c}m+|S|-1\\ m\end{array}}\right) ^{\frac{1}{2}} \sqrt{(m-1)!} \left( {\begin{array}{c}m\\ 2\end{array}}\right) . \end{aligned}$$

Similarly,

100 $$\begin{aligned} \Vert (\varphi _k^* + \varphi _{-k})(\varphi _k + \varphi _{-k}^*) \Phi ^{S}_{\le m} - \Phi ^{S \cup \{ k, - k \}}_{\le m+2} (e_k^* +e_{-k})(e_k + e_{-k}^*) \Vert \le c_{m}^{|S|} k_F^{1-d}, \nonumber \\ \end{aligned}$$

with some explicit constant $c_{m}^{|S|}$ depending only on *m* and |*S*|. Hence

101 $$\begin{aligned} \Vert H^{(1)} \Phi _{\le m}^S - \Phi H^B \Vert \le c_{m}^{|S|} N^{- \alpha } \sum _k |k| |{\widehat{v}}(k)|. \end{aligned}$$

### Proof

We have the identity

102 $$\begin{aligned} (\varphi _k \Phi -\Phi e_k)e^*_{q_1} \cdots e^*_{q_m} \Omega = \sum _{i=1}^m \varphi _{q_1}^* \cdots :\hspace{-0.25em}[\varphi _k, \varphi _{q_i}^*] \hspace{-0.25em}: \cdots \varphi _{q_m}^* \psi _0. \end{aligned}$$

Therefore, by (81) and the triangle inequality we have

103 $$\begin{aligned} \Vert (\varphi _k \Phi -\Phi e_k)e^*_{q_1} \cdots e^*_{q_m} \Omega \Vert&\le \sum _{i=1}^m 2c' k_F^{1-d} (m-1)!^{\frac{1}{2}} (i-1) \nonumber \\&= c k_F^{1-d} (m-1)!^{\frac{1}{2}} \left( {\begin{array}{c}m\\ 2\end{array}}\right) , \end{aligned}$$

where we put $c = 2 c'$. This proves (98). We see that $\varphi _k \Phi - \Phi e_k$ maps every element of the orthonormal basis (83) to a vector of norm at most the right hand side of (98). For an $m \times n$ matrix *T* we have $\Vert T \Vert \le \sqrt{n} \max _j \left( \sum _i |T_{ij}|^2 \right) ^{\frac{1}{2}}$, so we get

104 $$\begin{aligned} \Vert \Phi ^S_m e_k - \varphi _k \Phi ^S_m \Vert \le \dim ({\mathcal {D}}^S_m)^{\frac{1}{2}} c k_F^{1-d} (m-1)!^{\frac{1}{2}} \left( {\begin{array}{c}m\\ 2\end{array}}\right) . \end{aligned}$$

Using the identity

105 $$\begin{aligned} \Vert \varphi _k \Phi ^S_{\le m} - \Phi ^S_{\le m} e_k \Vert = \max _{0 \le n \le m} \Vert \varphi _k \Phi ^S_{n} - \Phi ^S_{n} e_k \Vert \end{aligned}$$

we obtain (99). Bound (100) is derived from (99) using $\varphi _k^* \Phi _{\le m}^S = \Phi ^{S \cup \{ k \}}_{\le m+1} e_k^*$ and the triangle inequality. Then, (100) implies (101). $\square$

### Lemma 8

Fix $K \in [2 \pi ,k_F]$ and let $S = \{ k \in (2 \pi \mathbb {Z})^d \setminus \{ 0 \} \, | \, |k| \le K \}$. For every unit vector $\psi \in {{\,\textrm{Ran}\,}}(\Phi _{\le m}^S)$ we have

106 $$\begin{aligned} | \langle \psi | H^{(2)} \psi \rangle | \le&(2k_F K+K^2)m + \frac{N^{-\alpha }}{2} \sum _{k \ne 0} |{\widehat{v}}(k)| (8 m (m+1)^{\frac{1}{2}} |C_k|^{\frac{1}{2}} +4m^2). \end{aligned}$$

If $\alpha < 1 - \frac{2}{d}$, the right hand side is $o (N^{1 - \alpha - \frac{1}{d}})$ for $k_F \rightarrow \infty$.

### Proof

If $p \in C_k$ with $|k| \le K$, then $|p+k|^2 - |p|^2 \le 2k_FK +K^2$. Since $\psi$ has at most *m* particles and holes created by $b_k^*$ with such *k*, the kinetic term is at most $(2k_F K+K^2) m$. For the other terms, we use Lemma 4:

107 $$\begin{aligned} | \langle \psi | d_{-k}(b_{-k}^*+b_k ) d_k \psi \rangle |&= | \langle \psi | (b_k^* +b_{-k}) d_k \psi \rangle | \nonumber \\&\le \Vert (b_k + b_{-k}^*) \psi \Vert \Vert d_k \psi \Vert \le 4m(m+1)^{\frac{1}{2}} |C_k|^{\frac{1}{2}}, \end{aligned}$$

and

108 $$\begin{aligned} | \langle \psi | d_{-k} d_k \psi \rangle | \le \Vert d_k \psi \Vert ^2 \le 4m^2. \end{aligned}$$

Then, we obtain (106) using the triangle inequality. The final claim follows from the upper bound in Lemma 3 and the assumption $d >1$. $\square$

We are now ready to finalize.

### Proof of Theorem 2

Let $f \in {\mathcal {D}}$. Then, for some $m \in \mathbb {N}$ and *K*, *S* as in Lemma 8 we have $f \in {\mathcal {D}}^S_{\le m}$. By Lemmas 5, 7, 8 and by (88) we have

109 $$\begin{aligned} E_N \le \frac{\langle \Phi f | H_N \Phi f \rangle }{\langle \Phi f | \Phi f \rangle }&= E_N^{(0)} + \langle f | H^B f \rangle + o (N^{1- \alpha - \frac{1}{d}}) \nonumber \\&\le E_N^{(0)} + c N^{1 - \alpha - \frac{1}{d}} \langle f | {\widetilde{H}}^B f \rangle + o (N^{1- \alpha - \frac{1}{d}}) . \end{aligned}$$

This proves that

110 $$\begin{aligned} \limsup _{k_F \rightarrow \infty } \frac{E_N - E_N^{(0)}}{N^{1 - \alpha - \frac{1}{d}}} \le c \langle f | {\widetilde{H}}^B f \rangle . \end{aligned}$$

Now optimize over *f* invoking Lemma 6. $\square$

## Footnotes

1 The series converges pointwise on the dense domain of all vectors obtained from $\psi _0$ by acting with a polynomial in $a_p^*$. By the sum of the series, we mean the operator closure of the expression defined first on the said dense domain. The same remark applies to other similar expressions below.

2 What is important for the proof of the lemma is that $\omega \cdot \omega ' \ge c$ for some constant $c>0$ independent of $\omega , \omega '$. Existence of such *c* follows immediately from the compactness of *D*, because clearly $\omega \cdot \omega ' >0$ for $\omega , \omega ' \in D$. However, inequality with explicit (and optimal) value of *c* can be deduced from a simple geometric argument, so we prefer to do that.

## References

[1] Lieb, E.H., Seiringer, R., Solovej, J.P.: Ground-state energy of the low-density Fermi gas. Phys. Rev. A **71**, 053605 (2005)

[2] Falconi, M., Giacomelli, E.L., Hainzl, C., Porta, M.: The Dilute Fermi Gas via Bogoliubov Theory. Ann. Henri Poincaré **22**, 2283–2353 (2021) 34720695 10.1007/s00023-021-01031-6 PMC8550787

[3] Giacomelli, E.L.: An optimal upper bound for the dilute Fermi gas in three dimensions. J. Funct. Anal. **285**(8), 110073 (2023)

[4] Huang, K., Yang, C.N.: Quantum-Mechanical Many-Body Problem with Hard-Sphere Interaction. Phys. Rev. **105**, 767 (1957)

[5] Lauritsen, A. B., Seiringer, R.: Ground state energy of the dilute spin-polarized Fermi gas: Upper bound via cluster expansion. J. Funct. Anal. 110320 (2024)

[6] Lauritsen, A. B.: Almost optimal upper bound for the ground state energy of a dilute Fermi gas via cluster expansion, preprint arXiv:2301.08005 10.1007/s00023-024-01450-1 PMC11799132 39926012

[7] Seiringer, R.: The Thermodynamic Pressure of a Dilute Fermi Gas. Commun. Math. Phys. **261**, 729–757 (2006)

[8] Lauritsen, A. B., Seiringer, R.: Pressure of a dilute spin-polarized Fermi gas: Lower bound, preprint arXiv:2307.01113

[9] Agerskov, J., Reuvers, R., Solovej, J. P.: Ground state energy of dilute Bose gases in 1D, preprint arXiv:2203.17183

[10] Graf, G.M., Solovej, J.P.: A correlation estimate with applications to quantum systems with Coulomb interactions. Rev. Math. Phys. **6**, 977–997 (1994)

[11] Bach, V.: Error Bound for the Hartree-Fock Energy of Atoms and Molecules. Commun. Math. Phys. **147**, 527–548 (1992)

[12] Bach, V.: Accuracy of Mean Field Approximations for Atoms and Molecules. Commun. Math. Phys. **155**, 295–310 (1993)

[13] Hainzl, C., Porta, M., Rexze, F.: On the Correlation Energy of Interacting Fermionic Systems in the Mean-Field Regime. Commun. Math. Phys. **374**, 485–524 (2020) 10.1007/s00220-019-03505-5 PMC7336250 32675828

[14] Benedikter, N., Nam, P.T., Porta, M., Schlein, B., Seiringer, R.: Optimal Upper Bound for the Correlation Energy of a Fermi Gas in the Mean-Field Regime. Commun. Math. Phys. **374**, 2097–2150 (2020) 32675828 10.1007/s00220-019-03505-5 PMC7336250

[15] Benedikter, N., Nam, P.T., Porta, M., Schlein, B., Seiringer, R.: Correlation energy of a weakly interacting Fermi gas. Invent. math. **225**, 885–979 (2021) 10.1007/s00205-023-01893-6 PMC10322800 37426631

[16] Benedikter, N., Porta, M., Schlein, B., Seiringer, R.: Correlation Energy of a Weakly Interacting Fermi Gas with Large Interaction Potential. Arch. Rational Mech. Anal. **247**, 65 (2023) 10.1007/s00205-023-01893-6 PMC10322800 37426631

[17] Christiansen, M.R., Hainzl, C., Nam, P.T.: The Random Phase Approximation for Interacting Fermi Gases in the Mean-Field Regime. Forum Math. Pi **11**, e32 (2023)

[18] Christiansen, M.R., Hainzl, C., Nam, P.T.: The Gell-Mann-Brueckner Formula for the Correlation Energy of the Electron Gas: A Rigorous Upper Bound in the Mean-Field Regime. Commun. Math. Phys. **401**, 1469–1529 (2023)

[19] Bohm, D., Pines, D.: A Collective Description of Electron Interactions: III. Coulomb Interactions in a Degenerate Electron Gas. Phys. Rev. **92**, 609 (1953)

[20] Pines, D.: A Collective Description of Electron Interactions: IV. Electron Interaction in Metals. Phys. Rev. **92**, 626 (1953)

[21] Gell-Mann, M., Brueckner, K.A.: Correlation Energy of an Electron Gas at High Density. Phys. Rev. **106**, 364 (1957)

[22] Sawada, K.: Correlation Energy of an Electron Gas at High Density. Phys. Rev. **106**, 372 (1957)

[23] Sawada, K., Brueckner, K.A., Fukuda, N., Brout, R.: Correlation Energy of an Electron Gas at High Density: Plasma Oscillations. Phys. Rev. **108**, 507 (1957)

[24] Luther, A.: Tomonaga fermions and the Dirac equation in three dimensions. Phys. Rev. B **19**, 320 (1979)

[25] Haldane, F. D. M.: Luttinger’s Theorem and Bosonization of the Fermi Surface, in Proceedings of the International School of Physics “Enrico Fermi”, Course CXXI: “Perspectives in Many-Particle Physics”, pages 5-30. North Holland, Amsterdam, (1994)

[26] Christiansen, M.R., Hainzl, C., Nam, P.T.: On the effective quasi-bosonic Hamiltonian of the electron gas: collective excitations and plasmon modes. Lett. Math. Phys. **112**, 114 (2022)

